# Two new species and one newly recorded species of *Elaphropeza* Macquart from Taiwan (Diptera, Empididae, Tachydromiinae)

**DOI:** 10.3897/zookeys.203.3284

**Published:** 2012-06-20

**Authors:** Jinjing Wang, Lili Zhang, Ding Yang

**Affiliations:** 1 Department of Entomology, China Agricultural University, Beijing 100193, China; 2 Key Laboratory of the Zoological Systematics and Evolution, Institute of Zoology, Chinese Academy of Sciences, Beijing 100101, China

**Keywords:** Diptera, Empididae, Tachydromiinae, *Elaphropeza*, new species

## Abstract

Previously 11 *Elaphropeza* species were known from Taiwan. The following two species of the genus *Elaphropeza* are described: *Elaphropeza flaviscutum* **sp. n.** and *Elaphropeza trimacula* **sp. n.** One species, *Elaphropeza plumata* Yang, Merz & Grootaert, is newly recorded from Taiwan. A key to 14 known species of *Elaphropeza* from Taiwan is presented.

## Introduction

*Elaphropeza* Macquartis a large genus in the subfamily Tachydromiinae traditionally placed in the family Empididae (Melander 1928; Steyskal and Knutson 1981; Woodley 1989; Yang et al. 2007; Cumming and Sinclair 2009) or assigned to the family Hybotidae (Chvála 1983; Sinclair and Cumming 2006; Chvála and Kovalev 1989; Shamshev and Grootaert 2007). *Elaphropeza* is very similar to *Drapetis* Meigen and was originally considered as a subgenus of the latter genus. It can be separated from *Drapetis* by the following features: occiput more convex; antenna not upturned; first flagellomere conical with lower margin as straight as upper margin; mesopleuron bare; hind tibia usually with 1-2 antero-dorsal setae (Collin 1961; Chvála 1975; Yang and Gaimari 2005; Shamshev and Grootaert 2007; Cumming and Sinclair 2009). It is distributed worldwide with 212 known species (Yang et al. 2007; Shamshev and Grootaert 2007; Grootaert and Shamshev 2012). The species from the Chinese mainland were reviewed by Yang and Gaimari (2005), and the Oriental species were reviewed by Shamshev and Grootaert (2007). Eleven species of the genus were known from Taiwan (Yang et al. 2007; Shamshev and Grootaert 2007). In this study three species including two new species of *Elaphropeza* are added to the fauna of Taiwan. A key to 14 known species of *Elaphropeza* from Taiwan is presented.

## Material and methods

The terminology follows Shamshev and Grootaert (2007). The types are deposited in Entomological Museum of China Agricultural University (CAU), Beijing. The following abbreviations for setae are used: acr–acrostichal, ad–anterodorsal, av–anteroventral, dc–dorsocentral, h–humeral, npl–notopleural, oc–ocellar, prsc-prescutellar, psa–postalar, pv–posteroventral, sc–scutellar, vt–vertical.

### Key to species of *Elaphropeza* from Taiwan

**Table:** 

1	Head black	2
–	Head yellow	*Elaphropeza xanthocephala* Bezzi
2	Hind tibia with 1-2 ad	3
–	Hind tibia without ad	*Elaphropeza trimacula* sp. n.
3	Hind tibia with 1 ad	4
–	Hind tibia with 2 ad	9
4	Mesoscutum entirely yellow	5
–	Mesoscutum patterned	8
5	Hind tibia with short rounded projection	6
–	Hind tibia with long pointed apical projection	*Elaphropeza calcarifera* Bezzi
6	Scutellum wholly brown or black	7
–	Scutellum brown medially	*Elaphropeza marginalis* Bezzi
7	Arista short, as long as basal three segments; acr and dc muliseriate	*Elaphropeza melanura* Bezzi
–	Arista very long, at least twice as long as basal three segments; acr biseriate, dc uniseriate	*Elaphropeza kerteszi* Bezzi
8	Arista not thickened; mesoscutum pattern not as below	*Elaphropeza pictithorax* Bezzi
–	Arista thickened with plumose pubescence; mesoscutum with two small lateral spots and one large mid-posterior spot	*Elaphropeza plumata* Yang, Merz & Grootaert
9	Mesoscutum entirely yellow	10
–	Mesoscutum patterned	12
10	Scutellum black at middle or entirely blackish	11
–	Scutellum entirely yellow	*Elaphropeza formosae* Bezzi
11	Scutellum black at middle; hypopleuron yellow, metapleuron brownish at upper part	*Elaphropeza scutellaris* Bezzi
–	Scutellum entirely black; hypopleuron black except narrow lower portion, metapleuron entirely yellow	*Elaphropeza flaviscutum*sp. n.
12	Hind tibia with indistinct, rounded projection	13
–	Hind tibia with long pointed apical projection	*Elaphropeza longicalcaris* (Saigusa)
13	Scutellum wholly black	*Elaphropeza lanuginosa* Bezzi
–	Scutellum black at middle	*Elaphropeza scutellaris* Bezzi

## Taxonomy

### Elaphropeza flaviscutum sp. n.

urn:lsid:zoobank.org:act:1A8DFF1F-A5BF-4D4A-82C6-D217BD5B2ACD

http://species-id.net/wiki/Elaphropeza_flaviscutum

Figs 1–2 5–7

#### Diagnosis.

Mesoscutum entirely yellow; scutellum entirely blackish; postnotum entirely blackish. Thoracic pleuron with only hypopleuron black except lower portion. Left surstylus large and broad; left cercus long with swollen apex.

#### Description.

Male. Body length 2.4–2.6 mm, wing length 2.2–2.5 mm.

Head black with pale gray pollinosity. Setulae and setae on head brownish yellow. Eyes contiguous on face. Ocellar tubercle with 2 oc and 2 short posterior setulae; 1 vt curved inward, slightly longer than oc. Antenna brown except scape and pedicel yellow; scape bare, shorter than pedicel; pedicel with circlet of blackish apical setulae; 1st flagellomere short, conical, 2.0 times longer than wide, short pubescent; arista long (4 times longer than 1st flagellomere), dark brown, short pubescent. Proboscis brownish yellow with blackish setulae; palpus yellow with blackish setulae and 1 blackish apical seta.

Thorax mostly yellow with thin pale gray pollinosity; mesoscutum lacking dark spots; scutellum and postnotum blackish, laterotergite yellow; hypopleuron black except lower portion, metapleuron without spot. Setulae and setae on thorax blackish; mesoscutum with sparse setulae; h absent, 2 npl (posterior npl longer), 1 sa, 1 psa, biseriate acr, uniseriate dc and 1 long strong posteriormost dc; scutellum with two pairs of sc (basal pair very short, about ¼ as long as apical pair). Legs yellow. Setulae and setae on legs blackish; fore coxa with 2 anterior setae at base, apically with 2 anterior setae; mid coxa apically with 3 anterior setae; hind coxa with 1 outer seta at apical margin. Fore femur 1.1 times as thick as mid femur, mid and hind femora subequal in thickness. Fore and mid femora each with 1 long thin pv at extreme base; mid femur with 1 preapical anterior seta; hind femur with 3 weak ad at base. Fore tibia apically with 1 av and 1 pv; mid tibia with row of short spine-like brown ventral setae, apically with 1 av and 1 pv; hind tibia with 2 ad, apically with 1 av. Hind tarsomere 1 without distinct ventral setae. Wing hyaline, veins dark brown, crossvein m-cu nearly vertical. Calypter brown with blackish setulae. Halter brown.

Abdomen mostly yellow with thin pale gray pollinosity; tergite 1 wholly membranous; tergites 2–3 brown, each with pair of subtrianglur lateral sclerites brown, medially linear and interrupted; tergite 4 broadest and tergite 5 narrow, blackish; tergite 5 anteriorly with large, separated, bare, blackish sclerite hidden within tergite 4; hypopygium dark brownish yellow. Setulae and setae on abdomen blackish except tergites 4–5 each with group of short squamiform black setae laterally, tergite 7 with row of long setae at posterior margin.

Male genitalia (Figs 5–7): Left epandrial lobe rather narrow in dorsal view, with surstylus large and broad in lateral view. Right epandrial lobe rather large in dorsal view, fused with apically narrowed surstylus. Left cercus long with swollen apex; right cercus rather short, about 1/3 as long left cercus.

**Figures 1–2.**
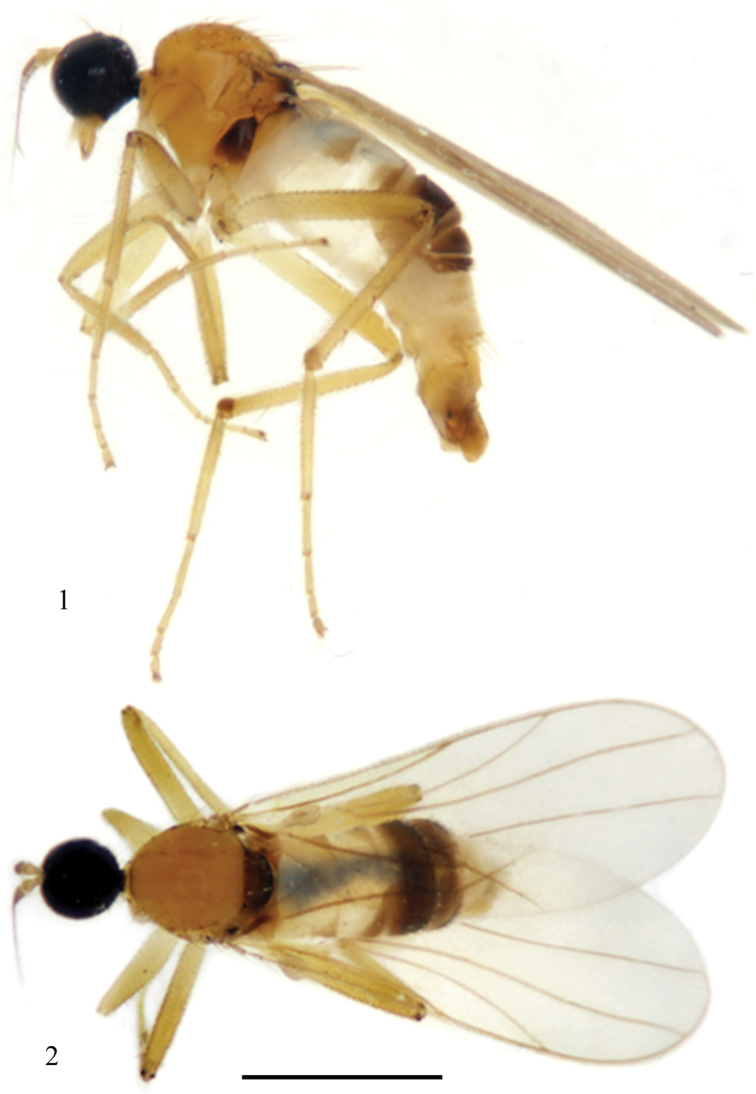
*Elaphropeza flaviscutum* sp. n. **1** adult, lateral view **2** adult, dorsal view. Scale bar 1 mm.

Female. Unknown.

#### Type material.

Holotype male, Taiwan, Nantou, Lianhuachi (120.8900E, 23.9260N), 2010. XI.11, Ding Yang. Paratype 2 males, same data as holotype. These specimens were collected from tropical forest by sweep net.

#### Distribution.

China (Taiwan).

#### Etymology.

The specific name refers to the yellow scutum.

#### Remarks.

This new species belongs to *Elaphropeza ephippiata* group, and is similar to *Elaphropeza scutellaris* Bezzi from Taiwan of China, but may be separated from the latter by the scutum entirely yellow, scutellum and postnotum entirely blackish, hypopleuron black except narrow lower portion and metapleuron without spot. In *Elaphropeza scutellaris*, the scutum usually has the indistinct vittae; the scutellum and postnotum are yellow with brownish spot in middle; the hypopleuron has no spot, the metapleuron is brownish at the upper part (Bezzi 1912; Shamshev and Grootaert 2007).

### Elaphropeza plumata

Yang, Merz & Grootaert

http://species-id.net/wiki/Elaphropeza_plumata

Elaphropeza plumata Yang, Merz & Grootaert, 2006: 575. Type locality: China: Guangdong, Zijing.

#### Diagnosis.

Arista thick with plumose pubescence. Mesoscutum with two small lateral spots and one large mid-posterior spot. Abdominal tergites 3–5 without short squamiform setae.

#### Specimen examined.

1 female, Taiwan, Jiayi, Shuisheliao (120.6595E, 23.6544N), 1165 m, 2010. XI. 16, Ding Yang. This specimen was collected from tropical forest by sweep net.

#### Distribution.

China (Guangdong, Taiwan).

#### Remarks.

This can be easily distinguished from other known species of the genus by the thick arista and unique marking pattern of the mesoscutum.

### Elaphropeza trimacula sp. n.

urn:lsid:zoobank.org:act:1EB36F18-11CA-4C67-B43D-911A6220D062

http://species-id.net/wiki/Elaphropeza_trimacula

Figs 3–4 8–10

#### Diagnosis.

Arista with distinct pubescence. Mesoscutum with three black spots. Hind tibia without ad. Left cercus rather large with 7 long strong apical setae.

#### Description.

Male. Body length 2.3 mm, wing length 2.5 mm.

Head black with pale gray pollinosity. Setulae on head yellow, setae brownish yellow. Eyes contiguous on face. Ocellar tubercle with 2 oc and 2 short posterior setulae; 2 vt curved inward, outer vt shorter than inner vt. Antenna dark brown except scape and pedicel yellow; scape bare, shorter than pedicel; pedicel with circlet of blackish apical setulae; 1st flagellomere long, conical, 2.4 times longer than wide, short pubescent; arista long (3.6 times longer than 1st flagellomere), dark brown, distinctly pubescent. Proboscis dark brownish yellow with blackish setulae; palpus yellow with blackish setulae and 1 blackish apical seta.

Thorax mostly yellow with thin pale gray pollinosity; mesoscutum with three blackish spots, median spot running through entire scutum and wider anteriorly; scutellum and postnotum black; pleuron with posterior portion (including hypopleuron, metapleuron and posterior portion of pteropleuron) black, mesopleuron and sternopleuron each with a blackish spot. Setulae on thorax yellow, setae brownish yellow; mesoscutum with short dense setulae; h absent, 2 npl (posterior npl longer), 1 prsc, 1 sa, 1 psa, acr and dc multiseriate and uniformly short; scutellum with two pairs of sc (basal pair very short, about ¼ as long as apical pair). Legs yellow except fore tibia and tarsus brown, mid tarsus and hind tarsomere 5 brownish yellow. Setulae and setae on legs blackish; fore coxa with 2 anterior setae at base, apically with 2 anterior setae; mid coxa apically with 3 anterior setae; hind coxa with 1 outer seta at apical margin. Fore femur 1.1 times as thick as mid femur, fore and hind femora subequal in thickness. Fore and mid femora each with row of short thin pv, and 1 long thin pv at extreme base; mid femur with 1 preapical anterior seta; hind femur with 3 weak ad at base. Fore tibia apically with 1 av and 1 pv; mid tibia with row of short spine-like black ventral setae, apically with 1 short av and 1 long pv; hind tibia without ad, apically with 1 av. Hind tarsomere 1 with 4–5 very short, irregular av. Wing hyaline, slightly uniformly tinged grayish; veins dark brown, crossvein m-cu oblique. Calypter brown with blackish setulae. Halter brown.

Abdomen dark brown with thin pale gray pollinosity; tergites complete except tergite 1 linear; tergite 3 relatively board, blackish; hypopygium blackish. Setulae and setae on abdomen blackish except tergites 3–5 each with group of short squamiform black setae laterally, tergite 7 with row of long setae at posterior margin.

Male genitalia (Figs 8–10): Left epandrial lobe rather narrow, with surstylus finger-like and apically curved inward in dorsal view. Right epandrial lobe rather large and broad, fused with surstylus of complicated shape. Left cercus rather long and large with 7 long strong apical setae. Right cercus very small (about 1/10 as long as left cercus), short finger-like.

**Figures 3–4.**
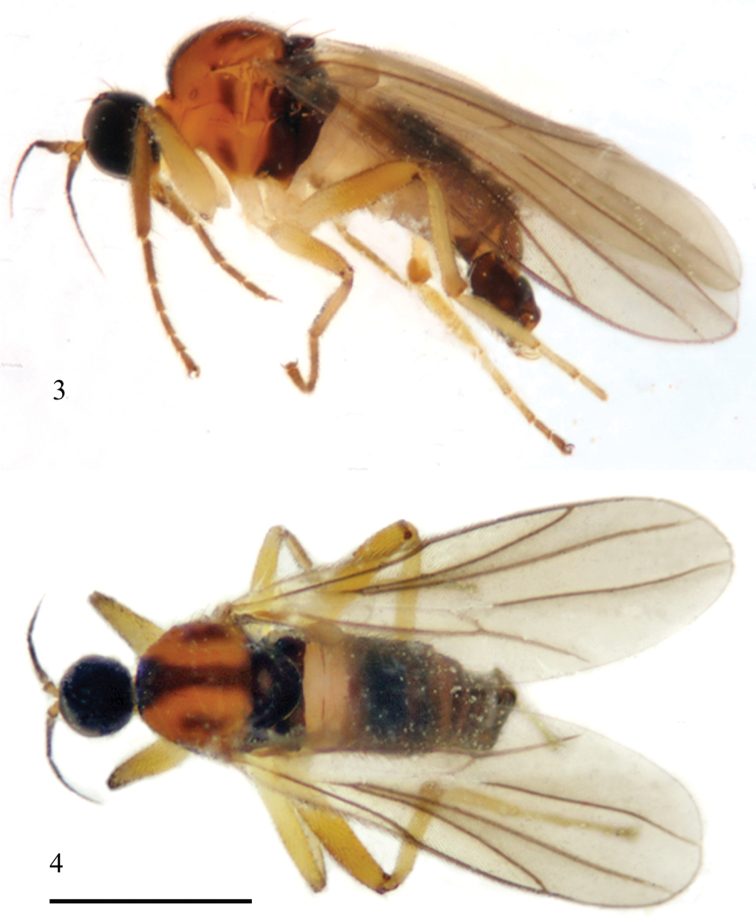
*Elaphropeza trimacula* sp. n. **3** adult, lateral view **4** adult, dorsal view. Scale bar 1 mm.

**Figures 5–7.**
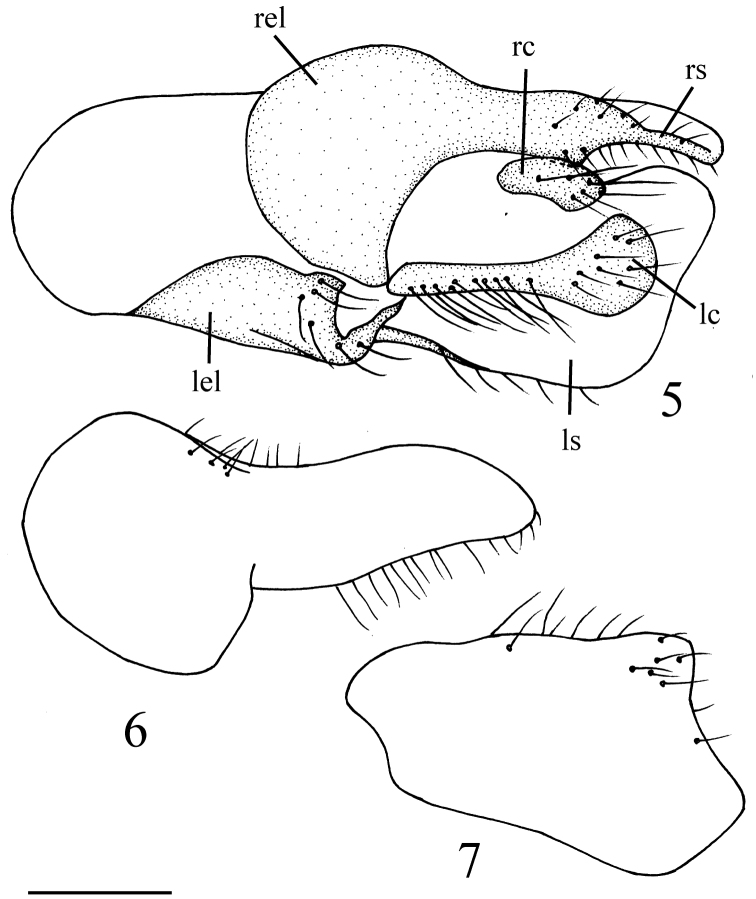
*Elaphropeza flaviscutum* sp. n. **5** male genitalia, dorsal view **6** right epandrial lobe **7** left surstylus. Scale bar 0.25 mm. Abbreviations: **lc** = left cercus; **lel** = left epandrial lobe; **ls** = left surstylus.

**Figures 8–10.**
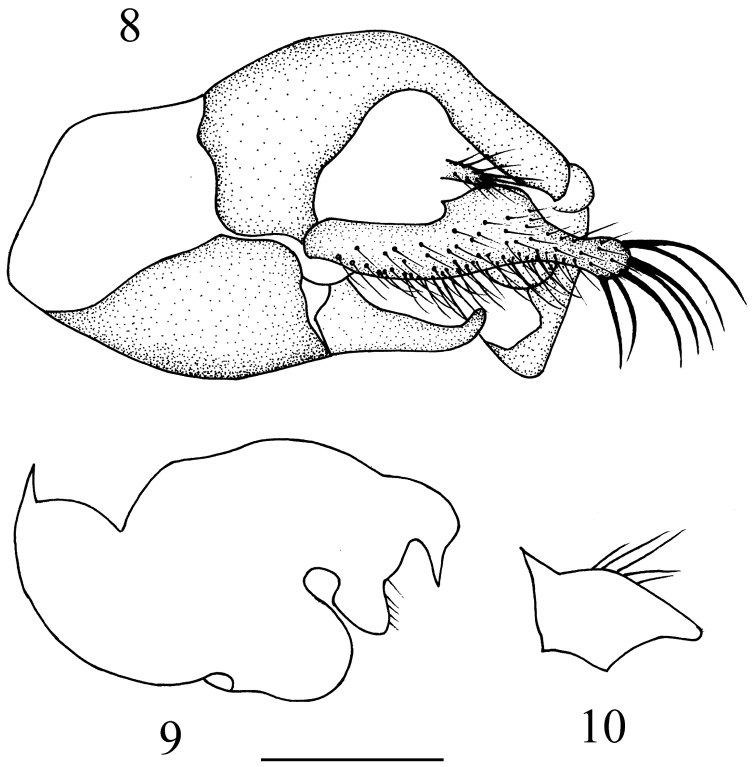
*Elaphropeza trimacula* sp. n. **8** male genitalia, dorsal view **9** right epandrial lobe **10** left surstylus. Scale bar 0.25 mm.

Female. Unknown.

#### Type material.

Holotype male, Taiwan, Taoyuan, Tamanshan (121.4507E, 24.7058N), 1620 m, 2011. VI. 14, Xiaoyan Liu. This specimen was collected from tropical forest by sweep net.

#### Distribution.

China (Taiwan).

#### Etymology.

The specific name refers to the mesoscutum with three spots.

#### Remarks.

This new speciesbelongs to *Elaphropeza biuncinata* group.In the key of Shamshev and Grootaert (2007), this species runs to *Elaphropeza acanthi* Shamshev & Grootaert from Singapore, but may be separated from the latter by the mesoscutum with three spots, the first flagellomere relatively long (2.4 times longer than wide), and arista with the distinct pubescence. In *Elaphropeza acanthi*, the mesoscutum has only one middle spot, the first flagellomere is relatively short (2.0 times longer than wide), and the arista is clothed in very short pubescence (Shamshev and Grootaert 2007).

## Supplementary Material

XML Treatment for Elaphropeza flaviscutum

XML Treatment for Elaphropeza plumata

XML Treatment for Elaphropeza trimacula
